# “*We call it Bokoa jwa tlhaloganyo*”: Setswana parents’ perspective on autism spectrum disorder

**DOI:** 10.3389/fpsyt.2024.1381160

**Published:** 2024-09-04

**Authors:** Neo Jeanett Melamu, Wandile Fundo Tsabedze, Petro Erasmus, Liezl Schlebusch

**Affiliations:** ^1^ Department of Psychology, North-West University, Mmabatho, South Africa; ^2^ Department of Psychology, University of South Africa, Pretoria, South Africa; ^3^ Division of Child and Adolescent Psychiatry, University of Cape Town, Cape Town, South Africa

**Keywords:** autism spectrum disorder, culture, parents, Setswana, caregiver

## Abstract

**Introduction:**

There is a dearth of knowledge in South Africa about the incidence, prevalence, and effect of autism spectrum disorder (ASD). Consequently, national autism data is outdated, and World Health Organization (WHO) prevalence rates are being used.

**Methods:**

This study focused on Ngaka Modiri Molema District to explore the cultural perspective of ASD in the Setswana culture from a parental or caregiver perspective, specifically those who attended the World Health Organization Caregiver Skills Training (WHO-CST) on ASD. This qualitative study used a phenomenological design and purposively sampled 6 out of 12 participants who wererecipients of WHO-CST. Semi-structured interviews, audio recordings, and field notes were used to collect data.

**Results:**

The study found five main themes: understanding autism, indigenous perceptions of ASD, ways of interacting with children living with autism spectrum disorder, creating a friendly environment and symptoms of ASD.

**Discussion:**

It was concluded that there is a lack of knowledge in Setswana culture about what ASD entails, and there are still some superstitious beliefs regarding ASD, resulting in late diagnoses. ASD studies with larger sample sizes, including medical professionals and policymakers, are recommended.

## Introduction

Autism spectrum disorder (ASD) is one of the many developmental disorders that need global attention. Cultural beliefs play a role in the understanding of ASD. Caregivers and parents of children living with autism need professional guidance dealing with ASD. Van der Merwe (1) reports that there is very little information available in South Africa about the incidence, prevalence, and effects of ASD. Uganda reported a 6.8:1,000 incidence rate of ASD (2). Unfortunately, owing to a lack of epidemiological research in South Africa, precise national data on ASD are unavailable in the region (North-West Province). According to the World Health Organization (WHO), international prevalence rates are presently being utilized in the South African context (3). The WHO has identified ASD as an important public health issue across global mental health services (4). ASD is more likely to be diagnosed at a late stage of development in low- to middle-income countries (5–8). Late diagnosis of ASD might be caused by a lack of knowledge, non-affordability to take the child to a specialist (psychologist, pediatrician, speech therapist, or others), and no awareness of ASD in that community (9–12). These problems are common in low- to middle-income countries with inadequate health and social care systems (13–15). ASD in Setswana culture is known as *bokoa jwa tlhaloganyo*, loosely translated as “mental condition”.

Abubakar et al. (16) and Abubakar et al. (17) state that the general absence of acceptable services and the low level of educational and medical infrastructure in Africa are some of the key practical and ethical challenges in recognizing children living with ASD. The lack of understanding about ASD is evident not only in the general population of Africa but also among those in the medical profession. Currently, there are no medical diagnostic tests for ASD; the medical practitioner looks at the child’s developmental history to make a diagnosis (17–19). A seminar presented by Bakare and Munir (20) in Nigerian psychiatrists and pediatricians who were asked about the roots of ASD implies that there is still a need for psycho-education about ASD in a cultural context.

As an illustration, Grinker et al. (21) claim that ASD is underdiagnosed in South Korea, South Africa, and other parts of Africa. They further report that it is due to ASD, which is generally not reported in clinical or academic records. Service availability is low in many countries (1, 22–24). This could be due to guardians’ failure to seek professional treatment. It is crucial to remember, however, that in some communities, cultural stigma and fear may be powerful enough to prevent a child’s caregivers from disclosing their special needs to healthcare professionals. Because of the stigma associated with having a child living with a developmental disorder, parents from other cultures (such as the Setswana culture) may believe that their child will have less access to services, will no longer be eligible to attend the parent’s preferred school, and will feel isolated from their extended family or community. In other words, the traditional environment has a significant influence on how an autism diagnosis is interpreted and how it affects people.

Qian et al. (25) and Grinker and Cho (13) report the unwillingness of South Korean parents to admit or accept their children’s autism diagnosis. They feel the need to define their child as “normal”; when that is not possible, there is a stigma attached to it. A parent of a child with autism might be concerned that other parents will prevent their children from playing collectively and that their household will become a source of gossip, which may also be evident in the current study. Furthermore, whatever the child endures may also happen to the parent (5, 26–29). For example, qualitative research conducted in Cape Town, South Africa, by Guler et al. (30) shows that caregivers expressed feelings of isolation, secrecy, and shame when their child was mislabeled as “naughty” by family members and the community. In Western countries, there is a high level of awareness of autism among health professionals (13). This level of understanding, however, does not exist in all societies, particularly in South Africa (31–33). Therefore, this study aims to explore the perspective of Setswana parents who attended the WHO-CST.

Participants for the study were parents of children living with ASD who had attended the World Health Organization Caregiver Skills Training (WHO-CST); this program was conducted during 2019–2020 in the Ngaka Modiri Molema District, specifically in Mahikeng area, and its international partners for families whose children have developmental delays or disabilities, including ASD. The program was implemented by a multi-disciplinary team (educational psychologist, research psychologist, clinical psychologist, psychiatrist, nurses, pediatricians, social workers, WHO Technical Officers, and WHO-CST Consultants). This team plays a significant role in ensuring that the WHO-CST program is delivered. Additionally, international specialists, caregivers, and family activists have an input in the delivery of WHO-CST. It is family-centered in nature to ensure collaborative care in a chain of social, educational, and healthcare services for children and their families. This program is extensively administered in many locations, such as the North-West Province.

**Figure 1:**
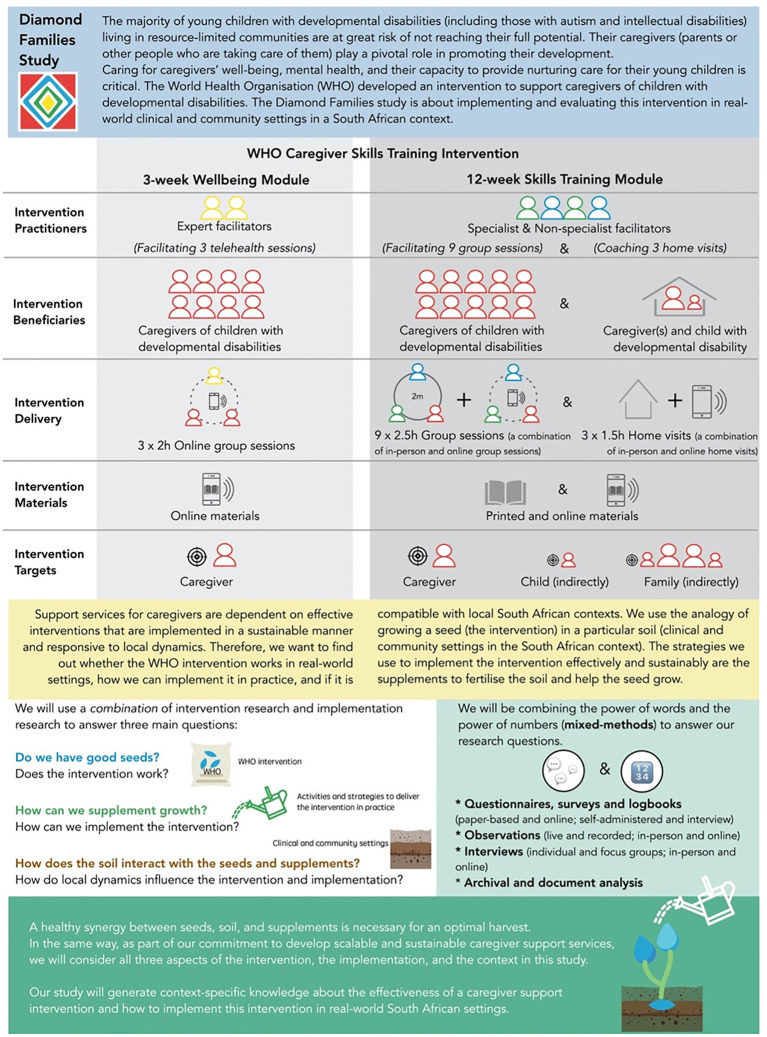
Original proposed protocol to field test the WHO CST Programme in South Africa.

The WHO-CST is for those who look after young children with developmental difficulties. This training program is intended to train non-specialist community facilitators to provide the intervention and was specifically created with low-resource settings in mind, including South Africa. Free of charge, caregivers attend a 3-week caregiver wellbeing module in three sessions, meeting once per week, at 2 h per session. The facilitators (specialist master trainers to non-specialist group facilitators) hold weekly meetings with caregivers during the 12-week program to discuss practical methods for fostering the child’s growth. The project also emphasizes the welfare of caregivers and attempts to lessen the stigma that is frequently associated with having a child with a handicap. More than 33 countries, including South Africa, are field testing the WHO-CST initiative. The National Department of Social Development, Autism South Africa, Centre for Autism Research in Africa at the University of Cape Town, and the North West University have joined forces on the South African pilot project. **Table 1**; **Figure 1** show in detail the WHO-CST program, for instance, administration, how long the intervention is, and how it is structured.

**Table 1:** Original description of the WHO CST Programme field tested in South Africa.

**Focus**	Caregiver education, skills training, psychosocial support, caregiver-mediated intervention	
**WHO CST modules used in this study**	3-week Caregiver Wellbeing module	
	12-week Skills Training module	
**Intervention materials**	Facilitator guides (group sessions and home visits)	
	Participant booklets	
	Training materials (guidelines, training toy kit)	
	Implementation and adaptation guidelines	
	Monitoring and evaluation guidelines and forms	
**Content**	**3-week Wellbeing Module**	
	Session 1: Introduction and what is important to you	
	Session 2: Doing what is important to you even when there is stress	
	Session 3: Review of program and closure	
	**12-week Skills Training Module**	
	Session 1: Introduction and getting children engaged	
	Session 2: Keeping children engaged	
	Session 3: Helping children share engagement in play and home routines	
	Session 4: Understanding communication	
	Session 5: Promoting communication	
	Session 6: Preventing challenging behavior, helping children stay engaged and regulated	
	Session 7: Teaching alternatives to challenging behaviors	
	Session 8: Teaching new skills in small steps and levels of help	
	Session 9: Problem-solving and self-care	
	Optional modules: Minimally verbal children; comorbid conditions	
**Format and intervention delivery**	**Wellbeing Module**	
	Group sessions (one/two facilitators delivering the intervention to a group of 8 caregivers)	
	- Practice getting present	
	- Review of previous contents and home practice	
	- Discussion of a story	
	- Presentation of new content	
	- Plan for home practice	
	**Skills Training Module**	
	Group sessions (two facilitators delivering the intervention to a group of 8–20 caregivers)	
	- Wellness activity	
	- Review of previous contents and home practice	
	- Discussion of a story	
	- Presentation of new content	
	- Demonstration	
	- Roleplay	
	- Plan for home practice	
	Home visits (two facilitators visiting the individual family home)	
	- Review of home practice and goal setting (caregivers set their own goals for their child)	
	- Coaching (modeling skills, reinforcing strengths, providing immediate feedback)	
**Dosage**	**Wellbeing Module**	**Skills Training Module**
Duration (how long)	3 weeks	12 weeks
Frequency (how often)	3 sessions, meeting 1× per week	12 sessions, meeting 1× per week or biweekly
Intensity (how much)	2-h session (online)	2.5-h group sessions; 90-min home visits
Booster sessions	Not specified in the original intervention protocol	Optional, informal, structured as a peer support group, 1–2 months after intervention
**Location(s)**	Online platform; easily accessible community venue; family home	
**Caregivers**	Caregivers (>18 years) who have a long-term caring responsibility for a child with DD	
	Two caregivers per family can participate in the intervention	
**Children**	2–9 years, although age can be slightly adapted in settings. In the South African study, we will include children 2–11 years. Caregiver concerns about developmental delays, disorders, or disabilities but no formal diagnosis is required	
**Intervention team**	A team that delivers the intervention consists of two facilitators, with supervision overseen by more experienced facilitators and/or trainers, and the possibility to include facilitators-in-training as observers. The three levels are:	
	WHO CST Specialist Master Trainers and Supervisors (Diamond Master Trainers)	
	WHO CST Specialist Facilitators and Trainers (Emerald Facilitators)	
	WHO CST Non-specialist Facilitators and Trainers (Sapphire Facilitators)	
**Training and supervision structure**	Blended train-the-trainer and apprenticeship training modules	
	Direct supervision and peer supervision structures	
	In-person and/or online training	
**Supporting international policies and frameworks**	Comprehensive Mental Health Action Plan 2013–2020	
	Resolution on “Comprehensive and coordinated efforts for the management of autism spectrum disorders”	
	Global Strategy for Women’s, Children’s and Adolescents Health 2016–2030	
	Mental Health Gap Action Programme (mhGAP)	
	Nurturing Care for Early Childhood Development	
	The Convention on the Rights of the Child, the African Charter on the Rights and Welfare of the Child and the Convention on the Rights of Persons with Disabilities	
	Sustainable Developmental Goals	
**Supporting national policies**	The National Integrated Early Childhood Development Policy	
	The Children’s Act No. 38 of 2005, as amended	
	Framework and Strategy for Disability and Rehabilitation Services in South Africa 2015–2020	
	The White Paper on the Rights of Persons with Disabilities 2015	

Source: Diamonds Family Study: FHS015 Research Protocol (May 2020).

This course is intended for those who provide care for children between the ages of two and nine who have developmental delays or other disabilities, particularly in the areas of communication and social interaction. A formal diagnosis is not required for families to take part in the program. The training aims to improve caregivers’ ability to use everyday play and household routines as opportunities to develop their children’s social skills, activity participation, good behavior, and daily life skills while also enhancing caregivers’ general wellness. The WHO’s Caregiver Skills Training for Families of Children with Developmental Delays or Disabilities program is the basis for this course. The goal of the WHO-CST is to teach caregivers how to use ordinary play and household tasks as opportunities for improved engagement, learning, and growth. The training consisted of three individual home visits and nine group sessions. The WHO-CST aims to help caregivers develop skills that will enable them to manage their children living with ASD. The focus of the WHO-CST is to equip caregivers with psychosocial and educational skills to assist them in caring for children living with ASD and to help them understand ASD.

A study conducted in Uganda tested 1,169 Ugandan children aged 2 to 9 in the Kampala region, which is semi-urban and semi-rural, with eight children diagnosed with ASD (34). The authors claim that the uncorrected occurrence of ASD is 6.8 per 1,000 individuals. The current study is one of the few studies conducted in the Ngaka Modiri Molema region, specifically exploring Setswana caregivers’ understanding of ASD. It cannot be ignored that this region is dominated by Setswana-speaking families of approximately 88.29% (Ngaka Modiri Molema, profile, 2020). Unfortunately, precise national data on autism are not accessible in South Africa due to a lack of epidemiological studies. The WHO stated that international prevalence rates are now being used (3). Talantseva et al. (35) maintain that the international frequency of ASD is on the rise; the most up-to-date statistics for the US reveal that 1 out of every 68 children under the age of 8 has the condition in the US, where there is increased awareness, screening, and better access to healthcare facilities for ASD. Yet, little is documented regarding the occurrence of ASD in South Africa, and the clinical features remain unknown for this region (3). Therefore, the research is relevant because no research has been conducted in the Ngaka Modiri Molema region, which indicates a research gap.

The cultural beliefs, norms, and values of parents can negatively influence their views on ASD diagnosis and treatment (36–38). Considering the results from the study and existing literature, culture affects the individual’s way of thinking and how they view the world. It is evident from the findings that parents having a child living with ASD report that their children were prohibited from playing with others due to society’s superstitious beliefs about the nature of ASD. Moreover, Bunt et al. (31) and Hebert and Koulouglioti (36) reviewed the literature concerning parental beliefs about the etiology of autism. They discovered that parents believed that genetics, environmental factors, and events related to childbirth are contributing factors. Brewton et al. (39) also support the above statement, stating that etiological thoughts regarding ASD also have an impact on behavior. Parents’ decisions about therapies, treatments, and/or resources were influenced by their beliefs about what caused their child’s ASD. We explored the perspectives of Setswana parents or caregivers on ASD, investigated the role of Setswana culture in understanding ASD and explore Setswana culture’s influences on parents’ or caregivers’ perspective of autism. These parents or caregivers attended WHO-CST, which was implemented in the Ngaka Modiri Molema District in 2019–2020.

## Method

### Description of the setting of the study

The research took place in the Ngaka Modiri Molema District, which is one of four districts in South Africa’s North-West Province. The population of the district is made up of 94.34% Africans (893,000), 3.38% Whites (32,000), 1.56% Coloreds (14,800), and 0.72% Asians (6,840). Setswana is the most spoken language at 88.29%, followed by Afrikaans at 3.29% and Sesotho at 2.26%, respectively (40). In the current study, five (83%) participants were between the age of 33 and 37, and one (17%) participant was 40 years old (see **Table 2**). Beliefs and religion were not the focus of the current study. This further explains the researcher’s choice of the population; additionally, participants are more likely to consult traditional doctors before they consult the healthcare system. This setting further gives a clear picture that the Ngaka Modiri Molema District is mostly covered by rural rather than urban areas.

**Table 2:** Demographic profile of the sample (*n* = 6).

Respondent code	Participant #1	Participant #2	Participant #3	Participant #4	Participant #5	Participant #6
**Age**	33 years old	37 years old	35 years old	33 years old	40 years old	37 years old
**Gender**	Female	Female	Female	Female	Female	Female
**Marital status**	Single	Married	Married	Single	Married	Married
**Employment status**	Employed	Employed	Employed	Unemployed	Employed	Employed
**Living with**	Two children and a mother	Husband, two children, and nephew	Husband and daughter	Mother, sister, and son	Two sisters and a son	Husband and son
**Education**	Matric	Matric	Bachelor’s degree	Diploma	Matric	Matric

The thematic data analysis comprises five themes: (1) Understanding autism, (2) Indigenous perceptions of ASD, (3) Ways of interacting with children living with ASD, (4) Creating a friendly environment, and (5) Symptoms of ASD. See **Table 3**.

**Table 3:** Themes and sub-themes of the study.

Themes	Sub-themes
**Theme 1:** Understanding autism	Sub-theme 1: Developmental delays/neuro-developmental disorder Sub-theme 2: Mental condition
**Theme 2:** Indigenous perceptions of ASD	Sub-theme 1: Bewitched Sub-theme 2: Superstitious beliefs (curse) Sub-theme 3: Visit a traditional doctor
**Theme 3:** Ways of interacting with children living with autism spectrum disorder	Sub-theme 1: Playing music Sub-theme 2: Building routines
**Theme 4:** Creating a friendly environment	Sub-theme 1: Perform home chores
**Theme 5:** Symptoms of ASD	Sub-theme 1: Before 2 years of age Sub-theme 2: Unease

### Description of Setswana culture in relation to the current study

The population of the study was sampled from the North-West Province, Ngaka Modiri Molema District, Mahikeng. In the Setswana culture, ASD is related to witchcraft. In this study, participants acknowledged that when their children were diagnosed with ASD, they first did nothing about it and were perplexed and unsure of what to do because ASD is viewed as a result of witchcraft or disobedience to the gods. Additionally, in the current study, families have shown no support towards caregivers or parents who care for children living with ASD. During family gatherings, caregivers have experienced their children being discriminated against and stigmatized. This implies that there is less support from family members and more likely that cultural beliefs and norms and values influence it. The Setswana culture shows a gap of knowledge and no understanding of ASD. WHO-CST is trying to bridge the gap by psycho-educating Setswana caregivers and parents on how to care for children living with ASD.

### Participants

The study solely focused on the Batswana or Tswana population, and no cultural screening was used to identify the participants; however, participants had to identify as Tswana people. Therefore, only participants from the Setswana culture were included in the study, as this is the population of focus. They had to be older than 18 years of age. They had to have participated in the WHO-CST program. Their child had to have been diagnosed with ASD. As the research focuses on the cultural perspective on ASD, the participants had to have experience in caring for a child living with ASD. The participants had to be fluent in Setswana or English, as the informed consent form and the data collection were in these two languages (given to the participant based on their preference). Out of 12 families, only 1 family was excluded because they were not from the Setswana culture. The exclusion criteria were based on the ethnic group of the participants. Therefore, those who were not from Setswana culture were excluded.

### Process

Participants were recruited after obtaining ethical approval from the North-West University Health Research Ethics Committee (NWU-HREC; ethics number: NWU-00104–22-A1), and goodwill permission was granted by Master Facilitators of WHO-CST in Mahikeng, North-West Province. The researcher used the WHO-CST census, which was conducted in the Ngaka Modiri Molema District, to recruit. Twelve families participated in the WHO-CST. However, only six consented and were willing to participate in the study. A flyer was then posted to a WhatsApp group chat for participants before we proceeded to data collection.

## Data collection

### Semi-structured interviews

Semi-structured interviews were conducted. An interview guide was used in English and Setswana (see **Appendix 1**). The semi-structured interviews were conducted in person, and the interviews were audio-recorded and then password-protected. The semi-structured interviews were intended to gather subjective feedback from the parents or caregivers regarding their perspective on ASD in the Setswana culture. The motivation for including caregivers who participated in the WHO-CST is that it allowed the researcher to collect data from not just one but multiple caregivers’ perspectives on ASD. The semi-structured interviews were utilized to explain parents’ or caregivers’ experiences, feelings, and opinions. In this study, the information obtained from the semi-structured interviews helped highlight the common themes that were common among the Setswana-speaking parents or caregivers.

### Data analysis

The collected data were analyzed using thematic analysis by Braun et al. (41), and the six steps of data analysis were applied. The study examined the subjective perspectives and sense-making regarding ASD in the Setswana culture. We conducted semi-structured interviews and wrote field notes. All the collected data were transcribed. Three researchers (N.J., W.F., and P.E.) decided on codes and final themes. This was to ensure that there were no biases during data analysis. For data analysis, codes were created for similar quotes, and extracted themes and sub-themes, and we then reported after member checking. Member checking was done to ensure that the study’s accuracy, credibility, validity, and transferability were met. During the interview, information was restated or summarized to determine accuracy. Member checking was completed after the study was shared with all of the study’s findings with the participants who took part in the study. This was done after data analysis at the same venue where data were collected. This allowed participants to analyze the findings and comment on them critically. The participants confirmed whether or not the summaries reflect their views, feelings, and experiences. Then, there is an affirmation of accuracy and completeness; thus, the study is said to have credibility.

## Findings

Setswana quotes were translated to English for inclusion in the study, but the quotes were not altered and might have grammatical errors. For example, *ke tlhaloganya gore* means “I understand that” in English; *ngwanake* loosely translates to “my child” in English.

### Demographic information

The program has records of 12 families who attended the training in the North-West Province, Ngaka Modiri Molema District. One family was not Setswana; therefore, 11 families were the targeted participants of the study. Only six participants participated in the study, and they were all from Setswana culture and female. It must be noted that families had additional family members, such as other siblings. Participants did not discuss if any of their family members are living with disabilities (mental health issues and/or physical disabilities). However, it is more likely that some of their family members are living or experiencing mental health issues or physical disabilities but were not discussed during interviews. The marital status of participants was either married or single. In terms of occupation, five participants were employed, and one was unemployed. Their age group was between 30 and 40 years. Lastly, their educational background ranged from matric (Grade 12) to a bachelor’s degree. **Table 2** shows demographic information obtained from the participants.

### Theme 1: understanding autism

#### Sub-theme 1: neuro-developmental disorder

From the perspectives of caregivers and parents, ASD is a mental condition, which, in their mother language, Setswana, they call “*Bokoa jwa tlhaloganyo*”. Two related sub-themes emerged: developmental delays/neuro-developmental disorder and mental condition. The similarity between the findings and available literature is that autism is viewed as a neuro-developmental disorder that affects children of all ages. Parents or caregivers commonly notice ASD through repetitive movements and communication difficulties. The condition often differs in terms of its severity.

*Participant 2: What I understand about autism is that autism is a neuro-developmental disorder. It is a disorder on spectrum in the sense that it deals with mmmm … neuro-developmental form of thing. You know, a lot of people that look at my daughter, they will be like aaaah … she is normal because she doesn’t have any physical appearances, you know, like a person who got up or Down Syndrome people will be able to see it’s a Down Syndrome or an adult, so with ASD you hardly see it; you can’t really see it because it’s all in the brain or neuro-developmental.* [Coughing] *… Sorry, I got a bit of flu.*

*Participant 3: What I understand ‘ke gore’ is that … it’s a neuro-developmental disorder whereby it affects the mind, how the mind, how the brain grows. Sorry. It will affect the child (yalo) like that, whereby there will be some delays etc.*

*Participant 4: My understanding about autism … okay, fine, let me refer back to my son how I understood from him. Because I could not, or did not, understand autism after I had him, that’s when I met autism. He was delayed in everything; his milestone came in later months than other kids. He was doing things that are different from other kids. By that I mean he started teething late; he started to sit when he was about close to nine months, and that’s when he started to sit on his own. I asked my local clinic nurses, and they just said he’s just a normal child, he will catch up, but as months went on, I saw that my son had a problem, but I did not know what it was until he was two and a half years old with no speech at all. Uhmmm … he did not have speech; he was walking in a different manner, like ‘ha tsamaya o ne a phuthula matsogo’ when he was walking, he was straightening his hands. I thought he was struggling with balancing, or he was hypersensitive. Yahhhh, that is my understanding of ASD.*

#### Sub-theme 2: mental condition

Participants showed an understanding of what is ASD in their perspectives and experiences.

*Participant 1: ‘Ke tlhaloganya gore’ I understand that autism spectrum disorder is a mental condition that goes with developmental delays, and learners or kids who are autistic tend to delay to speaking and eating. They differ from one kid to another.*

*Participant 5: I understand that autism spectrum disorder is a mental condition associated with a lack of speech and an inability to maintain social relationships. I would say I never knew about ASD until I met my daughter; I realised that ASD does not have a fixed definition because it differs from one child to another.*

### Theme 2: indigenous perceptions of ASD

#### Sub-theme 1: bewitched

Participants believe that ASD is caused by witchcraft and not obeying cultural laws. There are no differences between participants’ beliefs on what causes ASD.

*Participant 1: ‘Batswana ba nagana gore’ Tswana people think that autism is caused by witchcraft (go nale). There are some rituals (tse osa di dirang) you did not perform. ‘O tshwanetse o ise ngwana ko mabitleng’ You should take the child to the graveyard to introduce your child to your ancestors, they are punishing you; hence, ‘ngwana asa bue’ the child is non-speaking.*

*Participant 2: Obviously, when you have someone who is different, or you are different, or it is something they cannot explain traditionally, they would say that you have been bewitched or the mother or father, one of the parents did something they were not supposed to, that is a taboo within your culture. People will be telling you that you need to try these traditional ways. You need to go to one of the traditional doctors to try and get a cure for this, especially if there is no other person who got that diagnosis. So yahhh … you are told that the child is bewitched, or you, as the mother, you are punished by God.*

*Participant 5: In Setswana culture, they believe that autism originates from bad spirits that occur due to the mother or father’s negligence of his/her culture; hence, the child starts to have difficulties.*

#### Sub-theme 2: superstitious beliefs (curse)

*Participant 1: They felt that because I am a born-again Christian, there are some rituals I did not do culturally, which resulted in the child being non-speaking. They never experienced that thing in the family, both families where I was born and where my mother was born, and even in the family I am marrying into, this thing is new to them. So it’s like, we did something, so it is sort of a curse or bad luck based on the relative’s jealousy and all that, all these spiritual things.*

*Participant 2: Uhmmm … at the beginning, it was very difficult because even myself, I have never experienced anyone having ASD, even in my immediate family or even extended family members. It was very … very difficult trying to understand how to cope with this new diagnosis you have never heard of before. So, it was … even stigma here you are, my daughter is still non-speaking. So, you know her not being able to speak things she needed, it was just trial and error. Sometimes she would be just screaming there because now we are in a different environment. People had a problem, everybody understands that. My daughter is ten turning eleven, so now everybody knows and understands even at home, their understanding now is better than when we started initially.*

*Participant 3: They kind of think that our kids are crazy, they need mental health, or maybe we did not do traditional things for them. I am sure more than ten people used to ask me where the father of the child was, maybe I was supposed to take the child to the father’s family, maybe there was some rituals that were supposed to be done and whatnot. Only 5 per cent of the 100, 95 do not really understand. It’s like they do not want to understand, it’s like they do not want to understand; they think we are just spoiling our kids, they are just spoiled brats, we do not want to reprimand them.*

*Participant 4: You know when you are pregnant neh (correct)?…(pause) there are dos and don’ts. Your grandmother will tell you do not do this; you will suffer what … what you know. I was like, maybe there is something that I ate that caused autism spectrum disorder, or maybe I went somewhere, maybe in the graveyard, where I was not supposed to go. Eish, those superstitious beliefs.*

*Participant 6: I can say it was difficult for me to understand this condition because I never knew about it before. It was difficult because my son had delays, and he was not speaking. Even at crèche, his behaviour was different from other children. Even people would say that there is a curse or something is wrong … this is a form of bad luck; hence, I am having an autistic child.*

#### Sub-theme 3: visit a traditional doctor

*Participant 1: ‘Bare o tlhabele badimo, o kope thuso’ they say you should make a ritual for the ancestors, to seek for help. Go to traditional healers, and they can ask ancestors what is wrong with the child.*

*Participant 2: Obviously, when you have someone who is different, or you are different or it is something they cannot explain traditionally, would say that you have been bewitched or the mother or father, one of the parents did something they were not supposed to, that is a taboo within your culture. People will be telling you that you need to try these traditional ways; you need to go to one of the traditional doctors to try and get a cure for this, especially if there is no other person who got that diagnosis. So yahhh … you are told that the child is bewitched, or you, as the mother, you are punished by God.*

*Participant 3:* [Laughing] *…Aaah, they want us to take our children to traditional doctors for or to help you know…. they think that our children are crazy. They want the children to be taken to a mental institution. The awareness is now spreading and now big, but it seems like people are giving a dead ear. They don’t want to understand, especially Tswanas. We still have a long way with them.*

### Theme 3: ways of interacting with children living with autistic spectrum disorder

Participants showed to be more creative and interactive with children. This shows that participants use their knowledge to create play routines.

#### Sub-theme 1: playing music

*Participant 1: I found my own way of dealing with this, since there was no help from my family, my immediate family. Since I am a musician by profession, so I use music to do whatever with my son.*

The participant further explained how she interacts with the child via music.

*In terms of when I am getting him some food when I am making him bathe when I am making him brush his teeth, I use some guide I make him sing.*

*Participant 2: That is their non-existent word in Setswana culture because we do not believe in therapy if you are going through a mental disorder or breakdown. It is something that is happening to you; your neighbour knows what is happening to you, or one of your family members knows what is happening to you. So, such words are non-existent. Even now, when people come to my house, and they find us maybe doing electro-therapy and mesotherapy, they don’t understand.’ Why are you playing with water?’, for example. ‘What are you doing? Why are those things packed like that? ‘It is still non-existent, but I believe with education, we will get to that point.*

*Participant 4: Uhmmm … I believe when a child surrounds himself with other kids, he will start to learn better. They advised me to take him to a local crèche, where he will start playing. Unfortunately, with our kids are different; they prefer their own space.*

#### Sub-theme 2: building routines

*Participant 5: In Setswana culture, I don’t think we have play, unless, I don’t know. But, as for me, I have a little corner in the house where we usually meet and perform some kind of play, as we have building blocks, teddy bears, and cooking aid toys. I asked her to tell me what she would like to do for the day. I listen to her, and we do the task for the day.*

*Participant 6: I don’t know much about play, but what I normally do is I teach him how to take care of himself. Even routines, he even reminds me to give him his medication when I forget. He really likes to sing a lot. We play morabaraba and sometimes video games.*

### Theme 4: creating a friendly environment

Participants ensure that the home environment is conducive to children, for instance, by doing house chores.

#### Sub-theme: perform house chores

*Participant 1: I always try by all means to create a friendly space for my child. Like I said, we always sing to make him to remember the words I used and the things I said. Then, we perform home activities together, like cooking, cleaning, and doing laundry.*

*Participant 2: We do … we have, like, catalogues. I cut, like, pictures of milk or cheese or those types of things. Every time when I give her a picture, I would point to the picture ‘milk’, she would know that this is milk and home chores, even like buying the blocks, legos, and cars with different colours. I would buy one colour if we are playing with the colour, it would be green, to make her understand that concept.*

*Participant 3: Uhmmm…. Okay, we try to have routines, but sometimes it does not really help because my son is that person; he does not want fake things, he does not want to play with cars, and even if you give him a book, he does not want to play. You know, when you try to make him to colour a book, maybe we do colours, you must just point or ask which colour is this. It’s difficult, my dear. As for me, I think I have a bit of a challenge when it comes to routines because I have time in a day to teach him how to do home activities and when we learn the alphabets, he’s verbal; he would want to play with a cell phone. It’s difficult to have a routine with him, and he would want Channel O, play with a cell phone, or even go shopping. He likes clothes, that is his thing. Other than that, it’s difficult.*

*Participant 4: I try to make him take instructions, and we do home activities daily. Uhmmm….like after we eat, actually before he takes food to other,family members, he clears the table and takes plates after we finish eating. In the morning, after waking up, he knows he goes to the bathroom and undresses himself. I also train him to be independent. We are not yet there, but we are trying.*

*Participant 5: I usually ask for her help when I do home chores. We usually cook together and clean the house because she prefers to clean. I always guide her on how to clean. For example, she knows we pack things accordingly, such as how to use a broom and how to use a mop to sweep the floor.*

*Participant 6: He likes to offer help, like washing the dishes, sweeping the dishes, and sweeping the floor. I also guide him on how to pack groceries and dish up for himself when I’m not around. He prefers to do things independently, like brushing his teeth.*

### Theme 5: symptoms of ASD

Participants were able to see symptoms, though they were not sure what such behavior meant in their children’s different stages of development.

#### Sub-theme 1: before 2 years of age

*Participant 1: When he was two, I realised that his development was totally different from the older brother, especially regarding his diet. So, when he was two years, I started noticing that he likes to close his ears, he likes to, uhmmmmm … suck his bottle filled with any type of food. I started going to the internet, which is when I realised that he was having signs.*

*Participant 2: It was in 2013, she was about four months. It was in February 2013, uhmm, because there are certain milestones that you will know, that a child is starting to start sitting or doing whatever. But when she started sitting for a week, she wanted to lie down. If we try to make her sit, she will slide. It was basically her not being able to stand if you were trying to make her stand. She would be wobbling, type of thing. And playing down and not sitting down or showing those symptoms you would expect for certain milestones she was supposed to have reached.*

*Participant 3: When he was still a baby, I think when he was about … you know when a child should start noticing his surroundings, like, smile when you are trying to play with him. Do other stuff like smile, just follow also. Again, he did not care whether you were just standing in front of him. He would just stay like that; whatever you would do to make him laugh, he wouldn’t smile or do anything. The other thing that he was doing, which was weird, was eight days. Imagine eight days—nobody would expect this. She is my first child, but then my sister had a child in the house, and other family members had children. I would see how they behave and stuff like that. In eight days, my child managed to hold a small vent bottle; he understood that he must hold it nicely and feed himself, which was strange. I explained that to the clinic, and they said kids are different, so he is probably unique. So, I noticed at an early stage before he was diagnosed, I kind of, like … I diagnosed him. I started researching his behaviour and all that, and I also followed studies in America and whatever he was doing was relating to autistic kids. I could understand, and when Dr Titus finally diagnosed him, and we also went to a child psychologist, so he was finally diagnosed when he was four years old, and he was diagnosed with autism.*

*Participant 4: I can say, yaaaaah … six months is when I saw something first.*

*Participant 5: I realised that my child had difficulties when she was about two years. She did things differently, so I saw that by two years, a child must be able to speak or show interest to interact with other people.*

*Participant 6: My mother told me that my son is different from other kids, and that’s when I started paying attention to him. ‘O ne a rata gotshamekaka di toy tsedi blue fela’ he liked to play with only blue toys. He was about one year and six months old when I realised that he had difficulties. But, the clinic said it’s normal for some children to prefer certain toys or colours, and he was officially diagnosed with ASD when he was three years and two months old.*

#### Sub-theme 2: unease

*Participant 1: Yes, I had concerns. It’s just that with my firstborn he was also not eating that much, but he was not a choosy person; he would eat anything. So, the sad part with this one is that the time he was eating, he could even eat meat, but suddenly everything changed. He only wanted to drink milk, and I was really concerned.*

*Participant 2: Yes, because when someone is sick, there must be a reason for being sick. If you have TB, if you have cancer, there has to be a reason. So, if you have … if you have … there has to be a reason, and it starts with what could have happened. It starts by, did I do something differently … why … this child … why? Why my daughter? Why? Why? What contributed to the child being sick? You understand, yes, initially I got questions sick, now I got a better understanding, and in that, it got nothing to do with what I did.*

*Participant 6: Yes, I had concerns about my son. Because I could see that he was doing things totally different from other kids, like, at crèche, they always complained that he would just stand up and scream. When they try to understand what’s wrong with him, he would beat other learners.*

## Discussion

This study represents the understanding of Setswana caregivers or parents of children living with ASD who attended the WHO-CST program. The first theme shows that parents do understand what ASD is. This understanding may be because parents do care for their children. This can be through self-taught care or attending programs such as WHO-CST. For instance, participants stated that they understood autism as a neuro-developmental disorder and a mental condition that differs from one child to another.

As defined by Perrotta (18), ASD is a disorder characterized by developmental onset that affects 1% of people across all age groups. It is associated with delayed speech and repetitive movement (23). It also affects how the brain works and differs from one child to another. It is associated with delayed speech and repetitive movement. Participants reported that they view ASD as “*Bokoa jwa tlhaloganyo*”, loosely translated as a mental condition associated with developmental delays. This is supported by Harrison et al. (22), who stated that the general population’s knowledge of ASD varies as some people view autism as a developmental condition, disorder, or mental disability. Based on the findings of the study, autism is viewed as a mental disorder and is associated with developmental delays. The available literature agrees with the study’s findings on how autism is viewed in terms of it being a mental disorder associated with developmental delay, particularly affecting motor skills and language. According to a qualitative study conducted by Kenny et al. (27), the results show that people use different terms to describe autism, usually according to their cultural understanding. Additionally, parental and caregiver understanding about an illness is a major emphasis of psycho-education, and children affected by the disorder benefit from their parents’ knowledge (28). Parents and caregivers should be rendered psycho-education in the African context.

African perceptions of causes of ASD are associated with being bewitched, as reported by the participants in the second theme of the current study. Literature states that cultural perspectives play a critical role in identifying, diagnosing, and treating developmental disorders such as ASD (1). ASD is viewed in certain cultures as a kind of divine punishment or a result of demonic spirits or witchcraft (33), and Setswana culture is not an exception. Based on the study findings, it is reported by participants that African perceptions of causes of ASD are that an individual is bewitched or did not obey some cultural laws; hence, the child is autistic. Thus, participants revealed that disobedience of one’s culture or bad luck is due to superstitious beliefs (curse). The cultural misconceptions of ASD that exist in African culture in South Africa are still ongoing. In Mali, ASD is ascribed to supernatural origins, such as a curse, a divine punishment for a disobedient spouse, a spirit possession, or having annoyed evil spirits (32). Based on the findings and available literature, it is evident that the cause of autism is ascribed to disobedience of one’s culture and an individual being cursed. The difference between the literature and the results is that the latter report that ASD is due to some rituals that were supposed to be done, while the former argues that it is believed to be due to spirit possession.

Performing a ritual or consult, it is evident that a significant number of the participants believed that traditional doctors have supernatural powers to treat disorders based on their responses. ASD is stigmatized in Africa, and traditional health practitioners are the first to be consulted for diagnosis and treatment because of the prevalent cultural perception of ASD (38). According to Kang-Yi et al. (37), parents, when asked if they still consult with traditional doctors, disclosed that they still do as they believe society can heal people, particularly children (30). Based on performing a ritual or consulting, participants reported that they have to visit a traditional doctor to consult about what is wrong with the child or perform a ritual for the ancestors to seek help. One participant reported that she was advised to go to Zion Christ Church (ZCC) so that they could pray for her child.

Regardless of how traditions and culture perceive ASD, participants reported that they use ways such as playing music and building their own routines to interact with their children living with ASD; these are reported in the third theme. These ways of interacting with children and others are from traditional songs. Bunt et al. (31) indicate that the therapeutic potential of music could be applied to the care of children with autism. To engage children in a two-way communication process, attempts have been made to penetrate and elicit conscious or unconscious answers from them using the persuasive force of sound for children with autism (31). Along with musical sounds like pitch and rhythm, the aspects of music can also be used to foster social interactions (33). It can provide access to a wide range of emotional traits in a way that bypasses language and cognitive processing. Participants reported that they use methods such as building their routines on how to bathe and brush their teeth. In order to interact with their children living with ASD, some participants play music for their children, whereas some parents use guidance on how to sing.

Moreover, by creating a friendly environment for their children, they usually create specific practices. A friendly environment for children living with autism is vital, as stated by participants in the fourth theme. According to Wolfberg (26), children living with autism are less likely to play with objects in a useful way if they are not given clear instructions. Additionally, children with ASD rarely create pretend play by altering objects, using dolls as agents or creating fictional things, people, and events (26). Based on the study’s findings, it is evident that to create a friendly environment for their children, caregivers usually create specific practices. One participant reported that she encouraged the child to sing in order to help him remember the words she used and things she said. The participants also perform home activities together with their children, like cooking, cleaning, and doing laundry. The similarity is that all participants had certain house routines that they used in order to help their children to learn and develop.

In the fifth theme, participants reported that they noticed the signs of ASD in their children before 2 years of age. Autism can be diagnosed as early as 18 to 24 months. At this age, characteristic symptoms of autism can be distinguished from normal development, other delays, and other developmental conditions (12). The participants reported that they were aware that their children had noticeable signs of ASD before they were 2 years old, but they did not know it was ASD specifically. One participant reported that the child had a developmental issue, which was not being able to sit. Another reported that their child could not follow facial expressions, such as smiling.

The initial reaction of parents living with children with autism, based on the participants’ responses, is unease. According to Locke et al. (10), parents were typically the first to become concerned about their children’s development. Multiple presentations, including delays in language or motor development, sensory-seeking behaviors (for instance, spinning toys), and challenging behaviors, were described, even though the behaviors raising early concerns frequently represented core ASD deficits (for instance, impaired social engagement, limited eye contact, and severe tantrums that cannot be easily pacified). Participants reported that they saw that their children had a problem but did not know what it was. Most of the participants reported concerns about what might be wrong with their children, such as delay in the developmental stages.

### Strengths and limitations of the study

The study explored the stories of the participants, which gave an in-depth analysis of themes and sub-themes. The study’s limitations were that it only explored caregivers from the Setswana culture; this implies other cultures need to be explored, given that South Africa is a diverse country with 11 cultures. The study focused on caregivers and parental perspectives only; experiences of family members need to be explored. Only female individuals participated in the study, and this leaves a question of where are the fathers of the children living with ASD.

## Conclusion

The findings show that before attending the WHO-CST, most caregivers had little or no knowledge about ASD. Most caregivers experienced the challenges of late diagnosis and have developed various coping mechanisms to address and manage their children’s condition. The study’s findings strongly imply that ASD is a significant issue in South Africa. It is apparent from the caregivers’ past experiences that culture plays a significant role in a person’s life. There is a lack of knowledge in Setswana culture about what ASD entails. The findings also show that there are still some superstitious beliefs, causing late diagnoses of ASD in children; thus, it is important to continue to study and aim to understand ASD with even larger sample sizes, including medical professionals and policymakers, to enrich and increase the conclusions drawn about caregivers of a child living with ASD. Early interventions, such as WHO-CST, are necessary for the African community, specifically in South Africa.

Additionally, to help caregivers manage the difficulty of caring for their children who have ASD symptoms and are diagnosed with ASD, relevant policy changes and successful implementation are required. Psycho-education for parents and caregivers is essential. The intersection of ASD and culture is an appropriate topic in today’s world, as South Africa continues to experience an increase in children living with ASD. Lastly, it is recommended that researchers should find innovative ways to formulate early diagnoses of ASD to provide more knowledge concerning therapy options. Professional healthcare workers (psychologists, occupational therapists, social workers, medical doctors, and others) should provide free services to caregivers of children living with ASD and educate them about ASD.

## Data availability statement

The dataset is not publicly available due to the agreement with the participants. Requests to access the datasets should be directed to wandile.tsabedze@gmail.com.

## Ethics statement

The studies involving humans were approved by the North-West University, Health Sciences Research Ethics Committee (HREC), ethical approval number NWU-001-04-22-A1. The studies were conducted in accordance with the local legislation and institutional requirements. The participants provided their written informed consent to participate in this study.

## Author contributions

NM: Conceptualization, Data curation, Formal Analysis, Investigation, Methodology, Writing – original draft, Writing – review & editing. WFT: Conceptualization, Methodology, Project administration, Supervision, Validation, Writing – review & editing. PE: Conceptualization, Methodology, Project administration, Supervision, Validation, Writing – review & editing. LS: Conceptualization, Writing – review & editing.
