# Supplementary material for: “We call it Bokoa jwa tlhaloganyo”: Setswana parents’ perspective on autism spectrum disorder
Source: Front Psychiatry. 2024 Sep 4;15:1381160. doi: 10.3389/fpsyt.2024.1381160 (PMC11408941; doi:10.3389/fpsyt.2024.1381160)
Supplement: Supplementary file 1 [file DataSheet_1.pdf]

Interview schedule

The interviews used in the study seek to understand the cultural perspective of autism spectrum disorder in the Setswana culture from a parental perspective. There is no right or wrong answer. This study will enable me to help other people with the same challenges they may be experiencing.

Your name will not be mentioned during the interview. What you tell me during the data collection process will be used to enlighten the Tswana culture about ASD. Please participate in the research until the end. There are socio-demographic and interview guiding questions. Please tell me about your experience by answering the following questions.

Socio-demographic data:

Date of discussion	
Respondent code	
Age	
Gender	
Marital status	
Employment	
Living with	
Education	

Discussion guiding questions:

1. What do you understand about autism spectrum disorder?
2. What perspective does the Setswana culture hold on (the nature of) autism spectrum disorder?
3. What perspectives does the Setswana culture have on the causes of autism spectrum disorder?

4. What approaches does Setswana culture advocate for managing autism spectrum disorder?
5. How does Setswana culture define play for children living with autism spectrum disorder?

Probing questions

1) How does your family feel about you having a child with autism spectrum disorder?
2) When did you realise your child had difficulties?
3) What home activity routines do you do to help your child learn and develop?
4) Have you had similar concerns about what caused your child's condition?

General probes

- Can you explain a bit more?
- Can you give me more details about what happened?
- What happened next?
- Can you explain why?

Thank you for participating in the study!

Setswana interview guide

Dipotso tsa therisano:

1) O tlhaloganya eng ka bokoa jwa tlhaloganyo (autism)?

1. Ke dintlha dife tsa Setswana ka ga tlholego ya bokoa jwa tlhaloganyo?
2. Ke dintlha dife tsa Setswana kaga sebakwa sa bokoa jwa tlhaloganyo?
3. Ke dintlha dife tsa Setswana kaga go mekamekana le bokoa jwa tlhaloganyo?
4. Goya ka setsosa Setswana, ke eng go tshameka kalafi ga bana ba baphelang ka bokao jwa tlhaloganyo?

Dipotso tsa tlhaloso

1.Ba losika ba ikutlw ajang ka ngwana wa gago a nale bokoa jwa tlhaloganyo?

2.O lemogile leng gore ngwana wa gago o nale mathata?

3. Ke tsefe di tiro-gae tsaka metlha tseo di dirang go thusa ngwana wa gago go ithuta lego tokafala?

4.Ao kile wa nna le kamego ka tsela e etshwanang kaga sebakwa sa bokao jwa ngwana wa gago?

Dipotso tsa tlhaloso tseditlwaelegileng

- O ka tlhalosa go feta moo?
- O ka fa tshedimosetso ka gase se etsagetseng?
- Go diragetseng morago?
- O ka tlhalosa lebaka?

Ke lebogela matsapa a go tsaya karolo mothutong!
